# Oleuropein Aglycone, an Olive Polyphenol, Influences Alpha-Synuclein Aggregation and Exerts Neuroprotective Effects in Different Parkinson’s Disease Models

**DOI:** 10.1007/s12035-025-05208-6

**Published:** 2025-07-24

**Authors:** Milo J. Basellini, José M. Granadino-Roldán, Pablo V. Torres-Ortega, Giulia Simmini, Jaime Rubio-Martinez, Silvia Marin, Graziella Cappelletti, Marta Cascante, Ana Cañuelo

**Affiliations:** 1 Department of Biosciences, Università degli Studi di Milano, Milan, Italy; 2 Department of Biochemistry and Molecular Biomedicine, Faculty of Biology, Universitat de Barcelona, Barcelona, Spain; 3 Departamento de Química Física y Analítica, Universidad de Jaén, Campus “Las Lagunillas” s/n, 23071 Jaén, Spain; 4 Departamento de Biología Experimental, Universidad de Jaén, Campus “Las Lagunillas” s/n, 23071 Jaén, Spain; 5 Department of Materials Science and Physical Chemistry and Institut de Recerca en Quimica Teòrica I Computacional (IQTCUB), University of Barcelona (UB), 08028 Barcelona, Spain; 6 CIBER of Hepatic and Digestive Diseases (CIBEREHD), Institute of Health Carlos III (ISCIII), Madrid, Spain; 7 Institute of Biomedicine of University of Barcelona (IBUB), University of Barcelona (UB), Barcelona, Spain

**Keywords:** α-synuclein aggregation, Parkinson’s disease, Synucleinopathy, Oleuropein aglycone

## Abstract

Α-synuclein aggregation is the pathological feature of several neurodegenerative disorders, including Parkinson’s disease. The aggregates can diffuse within brain areas, and their toxicity has been proven in both cellular and animal models. Given that, recent therapeutic strategies have been focusing on the identification of compounds able to promote the degradation of aggregates or, at least, to prevent the aggregation process. In this field, the use of natural-derived polyphenols has been proposed as a potential tool against α-synuclein pathology. On these bases, we tested the neuroprotective potential of oleuropein aglycone, an olive polyphenol, in two cellular and *C. elegans*–based models of Parkinson’s disease. The compound was effective in reducing the burden of early-aggregates pathology upon α-synuclein overexpression in neuroblastoma cells, as well as neutralizing both the extent and the toxicity of administered preformed fibrils. In addition, oleuropein aglycone administration was beneficial for healthspan and lifespan in animals overexpressing α-synuclein, improved motor defects, recovered dopaminergic neuronal loss, and reduced the extent of α-synuclein pathology. Finally, through molecular modelling simulations, we propose a model for the α-synuclein and oleuropein aglycone interaction, predicting a dynamic that involves early α-synuclein oligomers. Overall, our results support the neuroprotective potential of oleuropein aglycone against α-synuclein aggregation and toxicity and shed light into the molecular features of these mechanisms, suggesting that further studies should be performed to gain insight about the neuroprotective actions of this polyphenol in humans.

**Supplementary Information:**

The online version contains supplementary material available at 10.1007/s12035-025-05208-6.

## Introduction

Α-synuclein (α-syn) is a small, natively unfolded, cytosolic protein of 140 amino acids and 14 KDa, encoded by the *SNCA* gene located on the long arm of chromosome 4 (4q21.3–q22) [1]. Despite the exact role of α-syn having still to be totally understood, the known list of its interacting partners is abundant and includes lipid membranes, synaptic vesicles, SNARE complex protein, microtubule cytoskeleton, and proteins involved in calcium regulation and dopamine homeostasis [2], suggesting that α-syn’s main function could consist in the modulation of neurotransmission, working on vesicle release and trafficking. α-syn was found to be the major constituent of Lewy inclusions [3], proteinaceous bodies that characterize synucleinopathy-affected brains. Concurrently, the involvement of α-syn in synucleinopathies was proved in 1997, following the discovery of the A53T mutation in the *SNCA* gene as the cause of a familial form of Parkinson’s disease (PD) [4]. Under specific conditions, including genetic mutations, post-translational modifications, and an increase in its physiological levels, α-syn can self-assemble to form β-sheets which results in the formation of insoluble aggregates [5]. α-syn aggregation occurs in a stereotypic way and follows a first-order kinetics: firstly, monomeric α-syn molecules participate in the formation of early multimeric species which associate themselves forming protofibrils and finally evolve into mature, insoluble fibrils [6, 7]. In this process, multimers or protofibrils can act as seeds capable of promoting and accelerating the conversion rate of physiological monomeric α-syn into aggregates [8]. Abnormal forms and aggregation of α-syn are a major hallmark in the pathogenesis of PD, and targeting α-syn misfolding and aggregation is considered a compelling therapeutic strategy to prevent neurodegeneration in PD [9, 10].

In recent years, there has been an increasing interest in finding molecules that prove efficient in preventing α-syn aggregation and fibrillation but are also capable of reducing the toxicity of preformed aggregated species. In this sense, many studies are focusing their attention on natural polyphenols present in food as important molecules to counteract amyloid aggregation and aggregate toxicity in different aging-associated diseases, including neurodegeneration [11]. Extra virgin olive oil (EVOO) is the major source of fat in the Mediterranean diet, which has been reported to exert different beneficial effects on health. Both the Mediterranean diet and EVOO consumption have been associated with longevity and with the ability to counteract the onset of neurodegenerative disorders [12], and it has been suggested that EVOO polyphenols could be among the main determinants of the beneficial effects of this diet. The main phenolic subclass in EVOO is represented by secoiridoids, which are present exclusively in plants belonging to the family of Olearaceae, which includes *O. europaea* L. [13]*.* The most abundant secoiridoids in olive oil are oleuropein aglycone (OA) and ligstroside, which are enzymatically transformed into hydroxytyrosol and tyrosol, respectively, in the gastrointestinal tract upon digestion [14].

In previous studies, different phenolic compounds contained in EVOO have been shown to exert beneficial effects on learning, memory, and cognitive impairment [15, 16]. In this sense, recent studies have focused on these compounds as potential inhibitors of amyloid aggregation, with tyrosol, hydroxytyrosol, and OA being the main phenolic compounds investigated due to their strong antioxidant capacity that has been shown to protect cells from oxidative stress and modulate different cellular and tissue processes [16–18]. These olive phenolic compounds have been reported to exert beneficial effects in amyloid neurodegenerative diseases [19], and it has been suggested that OA, in particular, may play a protective role by acting at different levels in the pathogenesis of PD. In this sense, recent studies have explored the effects of OA in α-syn misfolding and aggregation by using different approaches and experimental models [20–23].

The model organism *Caenorhabditis elegans* (*C. elegans*) presents interesting advantages to investigate the effects of olive polyphenols as neuroprotective candidates in different neurodegenerative disorders. Despite its simplicity, it presents tissues and organs like those in higher animals such as muscles, epidermis, gastrointestinal tract, gonads, and nervous system. In this sense, basic neurological pathways are highly conserved between invertebrates and mammals and around 50% of the human genome has recognizable orthologs in these nematodes. Furthermore, there are different transgenic and mutant strains available in *C. elegans* that reproduce the main features of human neurodegenerative diseases such as PD, Alzheimer’s disease (AD), or Huntington’s disease [24–27]. In particular, *C. elegans* PD transgenic strains present some of the most typical signs of this disorder, like α-syn misfolding and aggregation, impaired motor performance, and dopaminergic neurodegeneration.

To gain insight into these bioactive activities, the aim of the present study was to assess the ability of OA to protect against cellular α-syn aggregation and neurotoxicity by using well-established *in vivo* and *in vitro* models of PD. Furthermore, to elucidate the mechanism of action of OA at the molecular level and to pave the way for the identification of effective compounds against α-syn aggregation, we performed molecular dynamics (MD) simulations as a novel approach to unveil the interactions between OA and α-syn early-aggregates.

## Materials and Methods

### Cell Culture and Treatments

Naive and *SNCA* stable transfected SK-N-SH cells were gently provided by Arianna Bellucci (University of Brescia, Italy). Cells were cultured in low-glucose DMEM supplemented with 10% FBS, 1% non-essential amino acids, and 1% penicillin/streptomycin antibiotic. α-syn overexpressing cell line was generated according to Navarria et al. [28] and maintained in complete medium supplemented with Zeocin 50 µg/mL as selection antibiotic. Differentiation was performed by seeding cells on 0.1 mg/mL poly-L-lysine-coated dishes or 10-mm-diameter coverslip glasses, at a density of 5 × 10^3^ cells/cm^2^; then, cells underwent 3 days of 10 µM all-trans-retinoic acid treatment followed by 3 days of 10 µM all-trans-retinoic acid + 80 nM 12-O-tetradecanoyl-phorbol-13-acetate (TPA) treatment, for a total of 6 days. OA was administered on day 6, at 10 μM and 25 μM, as suggested by cytotoxicity test, while 0.1% DMSO (v/v) was used as control. On day 7, cells were either fixed in paraformaldehyde 4% in PBS + 10% glycerol for immunofluorescence or collected to perform biochemical assays. α-syn expression levels in both cell lines were evaluated by western blotting (Supplementary material S1).

Oleuropein (Extrasynthese, France) was resuspended in sterile-filtered MilliQ water, and oleuropein deglycosylation was performed according to [29]. Briefly, a 10-mM solution of oleuropein in 310 μl of 0.1 M sodium phosphate buffer, pH 7.0, was incubated with 8.90 IU of β-glycosidase (Sigma-Aldrich) overnight at room temperature. Then, the solution was centrifuged at 16,000 × g for 10 min to precipitate the compound. The supernatant was collected to test the oleuropein deglycosylation yield by assaying the glucose released in the solution with the glucose (HK) assay kit (Sigma), which resulted in 10 mM, indicating the complete deglycosylation of oleuropein. OA was then resuspended in dimethylsulfoxide (DMSO) to a 25-mM stock solution, aliquoted, protected from light, and stored at −80 °C. Hoechst test was performed to assess compound cytotoxicity (Supplementary material S2).

### Synuclein PreFormed-Fibrils (PFFs) Production and Administration

Full-length human α-syn was produced in *E. coli* and purified as previously described [30]. α-syn PFFs were prepared as follows: 3 mg of monomeric α-syn was resuspended under sterile conditions in 600 μl of sterile filtered PBS and centrifuged at 200,000 × g for 45 min at 4 °C to remove impurities. Supernatant was collected and filtered with 0.22 µm filters. The obtained solution of monomeric α-syn, 5 mg/ml, was maintained at 37 °C under orbital shaking (1000 rpm) for 14 days and was then maintained at 37 °C under orbital shaking for 14 days. PBS was incubated under the same condition for control. After 14 days, newly formed fibrils were separated from remaining monomers by centrifugation (16,000 × g for 18 min at RT) and resuspended in sterile filtered PBS to a stock concentration of 500 μM. The successful formation of fibrils was demonstrated by thioflavin T assay (ThT) and by transmission electron microscopy (Supplementary material S5 and S6). PFFs 2 µM were administered to naive SK-N-SH cells for 18 h on day 6 of differentiation, plus either DMSO 0.1% or OA 25 µM. On day 7, cells were fixed and underwent immunocytochemistry.

### Cell Viability Assays

3-(4,5-dimethylthiazol-2-yl)−2,5-diphenyltetrazolium bromide (MTT) assay was performed to assess cell viability. Cells were incubated with PFF 2 μM for 2 h, to ensure fibril sedimentation and consequent cell exposure. Then, the medium was supplemented, without removing the PFFs, with either DMSO (0.1%), OA 10 μM or 25 μM, for a total of 18 h of treatment. Then, MTT assay was performed according to manufacturer instructions, and 570-nm absorbance was read at an Ensight® multimode plate reader (PerkinElmer).

### Proximity Ligation Assay and Immunocytochemistry

To detect α-syn early-aggregates in the neuroblastoma cell line, we performed proximity ligation assay (PLA). The protocol was previously established [31] and slightly adapted from manufacturer instruction. Briefly, PLA probes *minus* and *plus* (Duolink® kit, Sigma-Aldrich) were conjugated to 1 mg/mL α-syn antibody (SynS3062, Sigma-Aldrich; rabbit, polyclonal, directed to human α-syn, amino acids 111–132) and stored at 4 °C. To perform the assay, paraformaldehyde-fixed cells underwent a PBS washing step, and then saturation of non-specific sites was carried out by a 30-min incubation with either Duolink® blocking solution (for PLA protocol) or 5% BSA in PBS + 0.1% Triton X (for regular immunofluorescence). Subsequently, cells were incubated with α-syn-*plus* and α-syn-*minus* probes (1:100 in PLA Diluent) plus anti-α-tubulin antibody (T6074, Sigma-Aldrich; mouse monoclonal, 1:500) for 2 h at 37 °C. The amplification reaction was accomplished by serial incubation with the following: (i) ligase in Duolink® ligation solution for 1 h at 37 °C; (ii) polymerase in Duolink® amplification reagent plus the secondary antibody donkey anti-mouse conjugated to Alexa Fluor® 488 (Molecular Probes) for 2 h at 37 °C. Finally, nuclei were counterstained with Hoechst reagent (Molecular Probes; 1:5000, 10 min), and samples were mounted on glasses using Mowiol® + DABCO® mounting solution. The specificity for synuclein early aggregates of probe-conjugated SynS3062 was performed as previously described [31]. Negative controls were carried out omitting one of the two probes. For regular immunofluorescence, the primary antibodies mix was prepared in BSA 1% in PBS + 0.1% Triton X and contained anti-α-syn antibody (Syn211, Thermo Fisher Scientific; mouse monoclonal 1:500) and anti-α-tubulin antibody (ab4074, Abcam; rabbit polyclonal 1:300). The secondary antibodies goat anti-rabbit conjugated to Alexa Fluor® 488 and donkey anti-mouse conjugated to Alexa Fluor® 647 (Molecular Probes) were diluted 1:500 in BSA 1% in PBS.

### Image Acquisition and Quantification

Confocal images of at least 10 steps were acquired at a Nikon spinning disk microscopy with a 60× objective. Areas containing at least 20 cells were selected, and α-syn early-aggregates were quantified by means of FiJi software on z-stack overlay images. Thresholds were established by means of the “Analyse Particles” feature. For 3D reconstruction, images were imported into arivis Vision4D^®^ 3.6.0 software (Zeiss). The region of interest (ROI) of 60× acquisitions, to segment cells and exclude not-cell related items, was created using the “Transformation gallery > Crop” tool. The “Intensity threshold segmentation” pipeline was applied to reconstruct cell bodies and nuclei. Synuclein positive PFFs were reconstructed using the “blob finder” pipeline.

### *C. elegans* Strains and Growth Conditions

The *C. elegans* strains N2 and NL5901 (pkIs2386; unc-54p:: α-syn::YFP + unc-119(+)) used in this study were obtained from the CGC (Caenorhabditis Genetics Center). The UA44 strain (bal11;Pdat-1::α-syn, Pdat-1::gfp) was kindly provided by Dr. G. Caldwell, University of Alabama, USA. The strain NL5901 overexpresses α-syn::YFP within worm body wall muscle cells and also exhibits age-dependent mobility defects associated with α-syn::YFP aggregation, which can be easily monitored. The strain UA44 expresses α-syn::GFP under the control of the dopamine transporter *dat-1* promoter, causing an age-dependent neurodegeneration of DA. Worms were propagated at 21 °C on solid Nematode Growth Media (NGM) seeded with the *E. coli* strain OP50 [32].

### Treatment with OA

For *C. elegans* assays, OA (3,4-DHPEA-EA; oleuropein aglycone) (Toronto Research Chemicals) was dissolved in DMSO and added at its final concentration to NGM previously autoclaved and cooled to 50 °C. The media was immediately dispensed into Petri dishes that were kept protected from light and stored at 4 °C until use. A final DMSO concentration of 0.1% (v/v) was maintained in all experimental groups. All the experiments involving OA were always carried out in parallel with a control group that contained only DMSO (0 mM OA).

### Lifespan and Heat Stress Assays

Synchronous populations of the NL5901 strain were obtained by egg hypochlorite preparation and incubation of L1 larvae on fresh NGM plates seeded with OP50. Synchronized L4 larvae were transferred to NGM plates containing fluorodeoxyuridine (FUDR), supplemented with 1 mM OA or DMSO, and lifespan scoring was initiated on the first day of adulthood (L4+1) at 21 °C. Adult nematodes were transferred to new treatment plates every 4 days. The number of surviving animals was monitored daily until death. Nematodes were considered dead when they did not respond to a mechanical stimulus with a platinum wire or no pharyngeal pumping was observed. Animals that became desiccated on the side of the plate, died by internal hatching, or exploded were censored and incorporated as such in the analysis. An average of 110 nematodes were used per experimental condition. For the heat stress assay, nematodes were incubated for 3 h at 37 °C on adult day 3 and survival was monitored from day 1 after stress exposure.

### *C. elegans* Quantification of Aggregates and Thrashing Assays

The quantification of aggregates was performed as previously described [18]. Briefly, NL5901 animals were synchronized, and L1 larvae were incubated in NGM plates containing *E. coli* OP50 at 21 °C until they reached the L4 developmental stage. Afterwards, L4 larvae were transferred onto FUDR+OA supplemented plates, while NGM plates containing only FUDR+DMSO were used as control. At the 5^th^ day of adulthood, worms were mounted in a 5μl drop of 10 mM levamisole (Sigma) on a 3% agarose pad, covered with a 24 × 24-mm coverslip, and observed under confocal microscopy to visualize fluorescent α-syn inclusions. Confocal microphotographs were obtained with Leica TCS SP5 II, and equal adjustment of brightness and contrast on control and matched experimental images was done using confocal software LAS-AF. For a more accurate quantification of individual inclusions, z-stack overlay images were analyzed using ImageJ software. Aggregates, defined as discrete, bright structures with boundaries distinguishable from surrounding fluorescence, were counted for each animal in the head region. Also, fluorescence intensity of the same head region was quantified in each animal. Three independent experiments with an average of 30 worms per experimental condition were performed.

For thrashing assays, 8-day adult nematodes were placed in groups of 5 on M9 buffer and allowed to recover for 2 min. Afterwards, movement was recorded for 1 min under a Leica stereomicroscope equipped with a DMK31AU03.AS camera (The Imaging Source). The number of body bends per minute was obtained using the Worm Tracker plugin (wrMTrck) for ImageJ software. Non-treated N2 *wild type* animals were used as control. A total of 30–40 animals were counted for each experimental group.

### *C. elegans* DA Neuron Degeneration Assay

For neurodegeneration assay, the *C. elegans* transgenic strain UA44 (bal11;Pdat-1::α-syn, Pdat-1::gfp) was used as described previously [18]. Briefly, L4-staged animals were transferred onto FUDR+OA supplemented or FUDR+DMSO plates and were grown at 21 °C during the whole assay. Worms were transferred to fresh treatment plates every 4–5 days. On the 7^th^ or 13^th^ day of adulthood, nematodes were placed on glass slides with 10 μl of levamisole, and GFP was visualized under confocal microscopy to assess for dopaminergic neurons integrity. Confocal microphotographs were obtained with Leica TCS SP5 II, and equal adjustment of brightness and contrast on control and matched experimental images was done using confocal software LAS-AF. For a more accurate assessment of neuron degeneration, z-stack overlay images were analyzed. The integrity of the four cephalic (CEP) dopaminergic neurons was evaluated for neurodegeneration according to previously described criteria [33]. Briefly, individual neuron dendrites were assessed for the presence of blebs, breaks, irregularities, or removal and scored from 0 to 6 values: *Score 0*, no damage, “perfect” neurons; *score 1*, irregular (kinks, curves, etc.); *score 2*, <5 blebs; *score 3*, 5–10 blebs; *score 4*, >10 blebs and/or breakage removing <25% of total dendrite; *score 5*, breakage, 25–75% of dendrite removed; *score 6*, breakage, >75% dendrite removed. An average of 120 dendrites was analyzed for each experimental condition.

### Molecular Modelling of the Interaction of OA with α-syn

In contrast to previous studies on the interaction of α-syn with different ligands [22, 34–36], which run a docking calculation and afterwards perform an MD calculation on the best obtained poses, we decided to take full account of the inherent protein flexibility when interacting with OA by simulating the binding process. Hence, our methodology consisted of 3 steps: 1, randomly locating OA in the simulation box; 2, running very long MD simulations; and 3, analyzing potential binding sites through both a clustering process and an average binding free energy analysis.

#### System Preparation

The cryo-EM structure of α-syn fibril (pdb entry 6CU7),[37] was used as the starting structure to perform MD simulations. This structure is identified by an orthogonal Greek-key-like architecture, comprising two interwoven protofibrils, each containing five α-syn peptide chains (residues 38–97). Zou et al. [38] reported that three of these α-syn chains are the crucial nucleus for the formation of α-syn fibrils. Therefore, we opted for an α-syn trimer to investigate the interactions established with OA and its role in the destabilization of α-syn oligomers. The system was placed in a cubic box, filled with TIP3P water molecules [39] using the LeaP module of AMBER20 [40], in such a way that there was a minimum distance of 15 Å between any atom of the solute and the box walls. OA was randomly placed in the simulation box, in such a way that 4 different simulation systems were obtained with different initial positions for OA to increase the conformational space explored by the ligand [41]. During the solvation process, water molecules closer than 1 Å to any atom of the system were removed. LeaP was also used to add the necessary counterions in the positions of lower electrostatic potential to neutralize the system.

#### Molecular Dynamics Simulations

MD simulations were performed using the AMBER20 [40] suite of programs. For each of the 4 systems, the α-syn trimer was parametrized using the ff14IDPSFF force field, which improves the sampling of intrinsically disordered proteins [42], while OA was parameterized using gaff2 parameters [43]. Electrostatic interactions were treated using the particle mesh Ewald (PME) method [44], and a cutoff of 10 Å was set to compute non-bonded interactions. Charges for OA were obtained with the restrained electrostatic potential [45] at the HF/6-31G* level. Prior to performing MD simulations, the structure of the α-syn trimer was relaxed to eliminate possible steric clashes, following a multistep minimization procedure using the steepest descent method [46]. After minimization, a heating and equilibration protocol was followed, including a heating up to 300 K at a rate of 15 K ps^−1^ under the NVT ensemble, followed by a simulation in the NPT ensemble in order to equilibrate density. Both steps were carried out using specific optimized parameters described elsewhere [47, 48]. After the equilibration process, each system was submitted to a production run of 6 µs length. Calculations were done using the hydrogen mass partitioning scheme [49] together with the SHAKE algorithm [50], permitting an integration step of 4 fs.

#### Clustering

Clustering was carried out to identify diverse binding sites of the α-syn trimer recognized by OA. The process was carried out using the average linkage algorithm [51] by means of the cpptraj module of AMBER20. Thus, for each of the four OA initial random positions essayed, which will be named from now on trimer 1–4, the trajectory was superimposed to the first frame and waters were deleted. Clustering was carried out, focusing on the positions occupied by OA, using the sieve option with a step of 10 frames, and aiming to obtain 15 clusters. Clusters with a population greater than 20% were further analyzed. Thus, 2, 1, 2, and 1 clusters were selected for trimers 1, 2, 3, and 4, respectively.

#### Cluster Analysis

To rank the different binding sites found after the MD and clustering process, both the average binding free energy and hydrogen bonds established for each cluster were calculated. The MMGBSA [52] methodology, employing the parameters optimized by Feig et al. [53] (igb = 2) for the generalized Born method, was utilized to conduct average binding free energy computations along with their decomposition analysis. The nonpolar solvation component, represented by $\Delta G_{nonpolar}^{solv}=\gamma SASA+\beta$, where SASA denotes the solvent-accessible surface area computed using the LCPO method [54], employed *γ* = 0.0072 kcal/mol·Å^2^ and *β* = 0 kcal/mol. MMGBSA calculations were executed using the MMPBSA.py software [55]. Hydrogen bonds were analyzed using default options with cpptraj. Visualization of the interactions between OA and the α-syn trimer was done with UCSF Chimera [56] and Maestro [57].

#### Statistical Analysis

Data are expressed as mean values ± SEM of at least three independent experiments. Statistical comparisons between the different experimental groups and their corresponding controls were made with Student’s *t*-test or log-rank test, unless otherwise specified, accepting *p* < 0.05 as the level of significance, using GraphPad Prism 6 software [58].

## Results

### OA Administration Significantly Decreases Oligomeric α-Synuclein Pathology in Neuroblastoma Cells

**Fig. 1:**
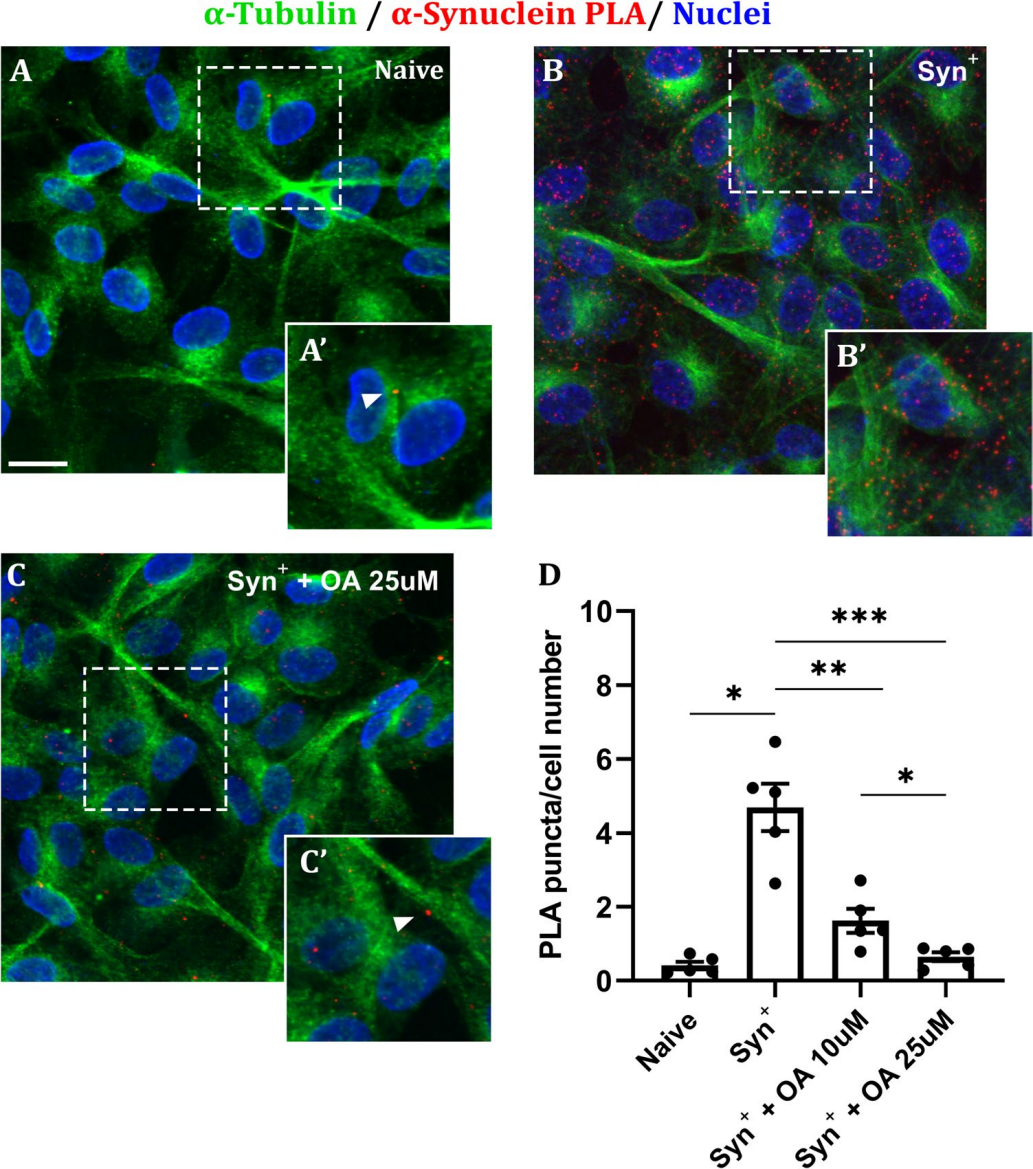
Effect of OA administration on early α-syn pathology in cells. **A** Naive SK-N-SH cells show little to no positivity to α-syn PLA (red signal). Green signal highlights α-tubulin; nuclei are counterstained in blue. **B** α-Syn overexpressing SK-N-SH cells naturally display an extensive amount of early-type α-syn aggregates (PLA signal, red dots). **C** The extent of α-syn pathology significantly falls after 24-h administration of OA 25 μM, as the number of red dots is notably decreased. Scale bar = 10 μm. Insets show 1.5× magnification of selected areas from the original images (white squares). **D** Quantification of α-syn aggregates. Each dot represents the average value obtained from multiple images containing at least 20 cells, collected from independent experiments, ± SEM. Naive = 0.41 ± 0.1; syn^+ ^= 4.7 ± 0.64; syn^+^ + OA 10 μM = 1.6 ± 0.33; syn^+^ + OA 25 μM = 0.65 ± 0.12. *p < 0.05; **p < 0.01; ***p < 0.001, according to one-way ANOVA

Α-syn overexpressing SK-N-SH cells were tested for the presence of early α-syn aggregates by PLA. Syn+ cells displayed, on average, a 10-fold greater α-syn pathology, compared to naive cells, evaluated as number of PLA puncta per cell (Fig. 1). Administration of 10 μM OA for 24 h caused a reduction of 65.4% in the number of aggregates per cell. By increasing the concentration of OA up to 25 μM, the extent of α-syn multimeric pathology was completely reverted to naive-like phenotype. The expression levels of total α-syn, evaluated by Western blotting, also appeared to be affected upon OA 25 μM administration (Supplementary material S3). In particular, α-syn levels were significantly reduced by 30% in treated cells (Syn+ OA 25 μM), compared to the control (Syn+, DMSO only), still remaining significantly greater compared to naive cells (2.06-fold). Cells were also tested for the presence of β-sheet enriched aggregates and phosphorylated α-syn, by means of the conformational antibody 5G4 and pS129 antibody, respectively. As shown in Supplementary Figure S4, 5G4 antibody provided no positive signal in either naive or Syn+ cells. Staining for pS129-synuclein revealed a diffuse pattern in both naive and Syn+ cells, thus likely to identify the presence of physiological, non-aggregated phosphorylated-synuclein. In addition, by ThS assay (Supplementary Figure S4.1), we confirm the absence of fibril-like aggregates in this model..

### OA Treatment is Protective Against Formation of Bigger Aggregates Upon Exogenous Synuclein PFF Administration

**Fig. 2:**
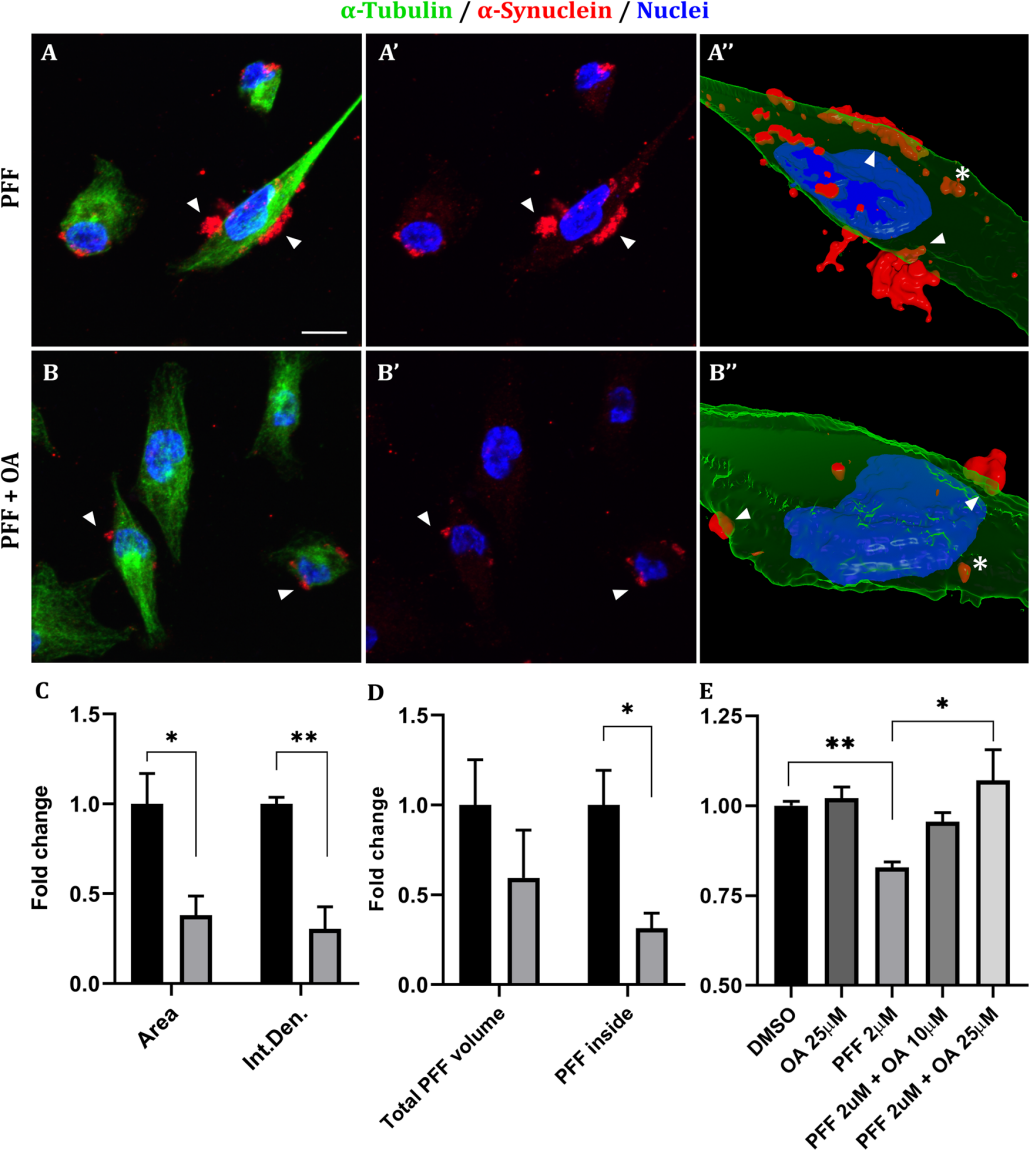
Effect of OA treatment on α-syn aggregates induced by exogenous PFF administration. **A** and **A’** Naive SK-N-SH cells exhibit great synuclein aggregates (arrowheads) after the administration of α-syn PFFs 2 μM. Red signal stains total α-syn, green signal highlights α-tubulin, nuclei are counterstained in blue. **B** and **B’** The extent of synuclein aggregates significantly decreases when PFFs are administered together with OA 25 μM. Scale bar = 10 μm. **A’’** and **B’’** 3D reconstructions (of **A** and **B**) through arivis Vision4D^®^ 3.6.0 software. Smaller aggregates (asterisks) appear to be internalized within the cells, while larger aggregates seem to be hooked to the membrane (arrowheads) and only partially internalized. **C** Quantification of α-syn aggregates evaluated as either total area covered by α-syn staining or intensity of red signal, normalized on nuclei. Columns indicate the mean of the values obtained by the quantification of at least 10 images collected by 3 independent experiments. Values are expressed as fold change of the control. Black columns = PFF 2 +DMSO; gray columns = PFF 2 μM + OA 25 μM. **D** Quantification of the volumetric relationship between cells and α-syn aggregates, evaluated as total area of PFFs interacting with the cells or the volume of PFFs internalized within cells. Analysis performed on the 3D reconstructions shown in A’’ and B’’. Columns indicate the mean of the values obtained by the quantification of 5 images collected by 3 independent experiments. Values are expressed as fold change of the control. Black columns = PFF 2 +DMSO; gray columns = PFF 2 μM + OA 25 μM. **E** MTT test to assess PFF toxicity and the beneficial effect of OA on cell viability. OA 10 μM administration led to a partial recovery from PFF-induced toxicity, while a complete recovery of cell viability was observed upon the administration of OA 25 μM. No effect on cell viability was observed upon the “OA 25 μM only” treatment. Data are expressed as fold change relative to the control (DMSO), showing means ± standard error of three independent experiments. *p < 0.05; **p < 0.01, according to Mann-Whitney test (**C**), one sample *t*-test and one-way ANOVA (**D**)

Naive SH-N-SK cells were incubated for 18 h with 2 μM α-syn-PFF together with either DMSO 0.1% or 25 μM OA and stained for α-syn (Fig. 2A, B). Aggregates were evaluated for both size and signal intensity and normalized on cell number. Both parameters displayed a significant reduction upon OA treatment (area = −62%; signal intensity = −70%) as shown in Fig. 2C. By 3D reconstructions (Fig. 2A’’, B’’) through arivis Vision4D^®^ 3.6.0 software, we revealed the spatial relationship between cells and PFFs. Specifically, while smaller aggregates appear to be internalized, larger aggregates seem to be hooked to the membrane and only partially within the cells. PFFs totally or partially internalized within cells were automatically segmented, and the volumes were calculated. As shown in Fig. 2D, the total volume of PFFs showed a notable (−41%) but not significant reduction upon OA administration. Nevertheless, when considering only the fraction of PFF volume internalized within cells, we observed a significant reduction (−69%, *p* = 0.012) in the treated cells. In addition, the fraction of cells presenting PFFs inclusion, of different extent, was significantly reduced by 52% (*p* = 0.027, not shown). Finally, cell viability assays indicate that OA administration results in the complete recovery of cell viability against the toxic effect exerted by Syn-PFF administration (Fig. 2E).

### OA Treatment Significantly Inhibits α-syn Aggregation and Improves Motor Activity in a *C. elegans* Model of PD

**Fig. 3:**
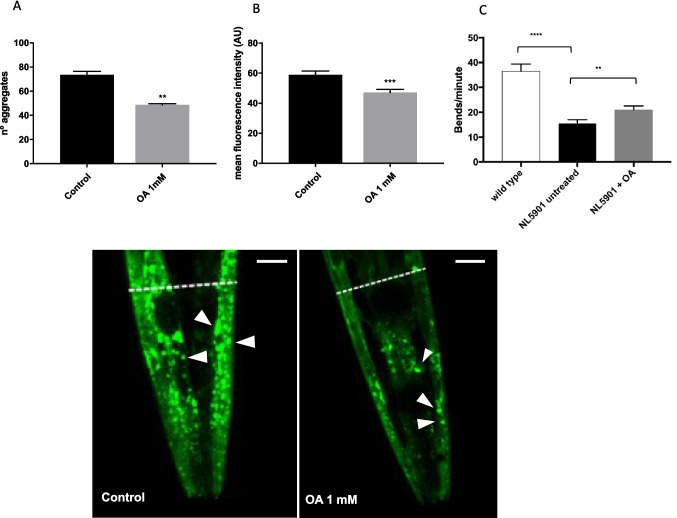
Effect of OA treatment on α-syn aggregation in a *C. elegans* model of PD. **A** Quantification of α-syn:YFP aggregates in muscle cells of 5-day adult NL5901 worms grown in the presence of 0 μM (control) or 1 mM OA. Results are represented as no. of discrete aggregates within the head region of each analyzed nematode (control 73.44 ± 1.71, vs. OA 1 mM 48.68 ± 0.5556; unpaired *t*-test **p = 0.0023); data are expressed as mean SEM of three independent experiments using a total of 90 nematodes per experimental condition. **B** Quantification of total body fluorescence in the same nematodes analyzed in **A**; unpaired *t*-test ***p = 0.0007. **C** Worm-thrashing analysis represented as bends per minute of N2 *wild-type* (white) and NL5901 nematodes untreated (black) and treated with OA 1 mM (gray); unpaired *t*-test **p < 0.01, ****p <0.0001. **D** Representative images of α-Syn::YFP aggregates in muscle cells of the head region of 5-day adult NL5901 nematodes obtained by confocal microscopy and Z-stack overlay (scale bar 10 μm). Untreated control (left panel) and OA 1 mM–treated (right panel) nematodes are shown, and the head region analyzed is delimited with a dotted line. Some of the aggregates are indicated by white arrowheads

To assess potential OA inhibition of pathological α-syn accumulation in a living organism, we used a well-established model of PD in *C. elegans*, the strain NL5901, which expresses human α-syn fused to the yellow fluorescent protein (YFP) under the control of the muscular *unc-54* promoter. In this strain, muscle expression of α-syn has been widely used to model protein misfolding diseases [25]. OA concentration at 1 mM was selected in *C. elegans* assays based on previous studies using OA and other olive polyphenols in *C. elegans* strains [18, 20]. To analyze the effect of OA in α-syn misfolding and accumulation, nematodes were incubated with or without the compound starting at the larval stage L4, and the number of inclusions was quantified at the adult day 5. We also measured total fluorescence intensity in the worm bodies to provide further validation to the aggregate quantification. Treatment with 1 mM OA resulted in a significant decrease in the amount of visible α-syn aggregates compared to untreated nematodes by an average of 24.7 units (OA 1 mM 48.68 ± 0.5556 vs. control 73.44 ± 1.71, unpaired *t*-test **p value = 0.0023) (Fig. 3A). Since we quantified individual aggregates in z-stack confocal images, our results are more accurate than those obtained only by total fluorescence intensity determination. In this sense, although our results analyzing total fluorescence in the same individuals also showed a slight reduction in α-syn fluorescence after OA treatment, the decrease compared to control was less prominent than the one observed in the aggregate quantification (Fig. 3B). To further clarify this aspect, we evaluated the level of α-syn protein expression in nematode extracts and confirmed that OA did not significantly reduce total α-syn:YFP levels compared to untreated controls (Supplementary material S9), suggesting that OA influence on α-syn aggregation is not due to a reduced expression of this protein.

The expression of α-syn in the body wall muscle cells in the NL5901 strain leads to movement impairments as compared to *wild type* nematodes [25]. Thus, we sought to analyze the effect of OA on motor activity in this PD strain by quantifying the thrashing rate at adult day 8, when the motor deficits are more clearly observed. Indeed, at this stage, the number of bends per minute decreased significantly from 36 in *wild type* adults to 15 in untreated NL5901 worms (Figure 3C). Interestingly, treatment with OA induced a slight but significant increase in the thrashing rate in NL5901 nematodes (15.5 vs. 20.97) (Figure 3C), suggesting a delayed α-syn-dependent motor decline in response to OA.

### OA Extends Lifespan and Improves Survival After Heat Stress in a *C. elegans* Model of PD

**Fig. 4:**
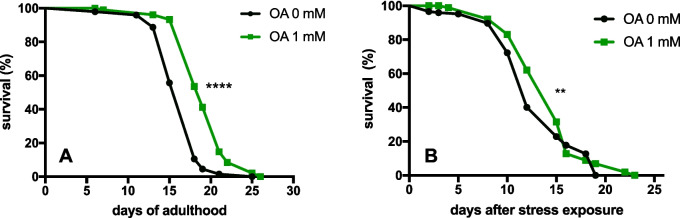
Effect of OA on lifespan and heat stress in a *C. elegans* model of PD. **A** Survival curves of NL5901 nematodes. Each curve represents two independent experiments, and survival is expressed as a percentage of the initial population per day. **B** Heat stress survival in the presence of 1 mM OA. At the third day of adulthood, NL5901 nematodes were exposed to heat stress for 3 h at 37 °C before monitoring survival (day 0). Table 1. Lifespan and thermal stress survival data after OA treatment. Statistical significance was calculated by log-rank test; differences compared to control ****p < 0.0001, **p = 0.0075. *n*, number of tested nematodes

Different olive polyphenols and extracts have been reported to extend the lifespan of *C. elegans.* In this sense, we have previously shown that tyrosol is able to significantly induce lifespan extension in both *wild type* and NL5901 *C. elegans* strains. Although a recent study has reported that OA treatment failed to exert significant effects on lifespan in *wild type* nematodes [20], OA effect on lifespan has not been tested before in a *C. elegans* model of PD. Considering the significant reduction of α-syn accumulation in muscle cells exerted by OA, we decided to evaluate if this treatment was also effective in extending the longevity of the NL5901 strain. Our results showed that treatment with OA 1 mM led to an increase of median lifespan by 10%, resulting in significantly different survival curves (*p < 0.0001; log-rank (Mantel Cox) test)) (Fig. 4A; Table 1).

**Table 1:** Lifespan and thermal stress survival data after OA treatment

**Lifespan**			**Thermal stress survival**		
Treatment	***n***	Median survival (days)	Treatment	***n***	Median survival (days)
Control (DMSO)	183	18	Control (DMSO)	147	12
OA 1 mM	191	20^****^	OA 1 mM	183	15^**^

Statistical significance was calculated by log-rank test; differences compared to control ****p < 0.0001, **p = 0.0075. *n*, number of tested nematodes

Lifespan and aging-related pathologies are strongly correlated to heat stress resistance in *C. elegans*; thus*,* we decided to monitor survival after stress exposure to further corroborate the lifespan results obtained before in this PD strain. Our results showed that OA was able to induce an increase in median survival after heat stress by approximately 16% compared to controls (Fig. 4B; Table 1). As is shown in Figure 4B survival curve, although the % survival in OA-treated nematodes appears lower at specific time points, the median age at which the population starts to decline is lower in the control groups compared to the OA-treated ones.

### OA Treatment Prevents Degeneration of Dopaminergic Neurons in a *C. elegans* PD Model

In the *C. elegans* UA44 transgenic strain, the eight dopaminergic neurons can be visualized due to GFP-α-syn linked to the dopamine transporter protein. α-syn expression and accumulation in dopaminergic neurons lead to age-dependent neurodegeneration that can be visualized and evaluated by fluorescence microscopy in this strain [59, 60].

**Fig. 5:**
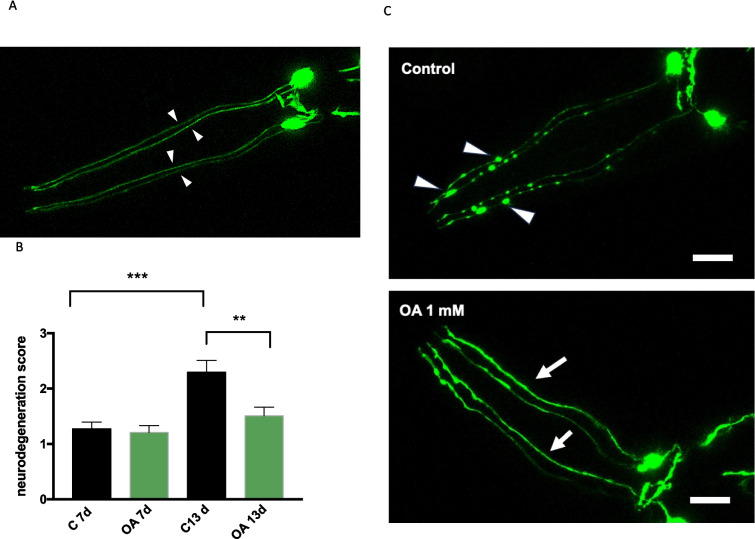
Effect of OA treatment on dopaminergic degeneration in *C. elegans*. **A** Representative image of a young adult UA44 nematode, expressing GFP-α-syn specifically in DA neurons. The four CEP anterior dopaminergic neurons, indicated with white arrowheads, are shown as an example of intact dendrite morphology (score 0), since at this age, dopaminergic neurons have not started to show degeneration. **B** Degeneration of the 4 anterior dopaminergic neurons was evaluated at 7 and 13 days of adulthood on z-stack overlay images obtained by confocal microscopy. Data represent the median score values (0–6) [33] obtained for each experimental group. An average of 120 dendrites were analyzed for each experimental condition. ***p value 0.0007; **p value 0.009, *U*-Mann Whitney test. **C** Representative images of 13-day-old UA44 nematodes grown in the presence of 0 μM (control) or 1 mM OA. Arrowheads in the control image (above) indicate typical blebs observed in neurodegenerating dendrites. In these particular dendrites, the damage would be evaluated as *score 3* (5–10 blebs). In the OA image (below), an example of typical *score 1* dendrites is indicated with white arrows in the image, (score 1: irregular, kinks, curves along the dendrite) (scale bar, 10 μm)

To determine if OA was able to affect the age-dependent neurodegeneration phenotype in this PD model, we analyzed morphological changes in the four CEP anterior dopaminergic neurons at two different adult stages. We adapted a previously described method based on scoring detectable changes in dendrites morphology [33]. As an example, 4 intact dendrites (score 0) are shown in image A (Fig. 5A). In untreated nematodes, our results showed an evident increase in dendrite neurodegeneration from adult day 7 to adult day 13. Although we did not observe differences in response to OA treatment at adult day 7 stage, in older worms (13 days), OA induced a significant delay in dopaminergic dendrites deterioration, with a lesser presence of blebbing and irregularities along the prolongations compared to dendrites in control nematodes (Fig. 5B, C). These results suggest that OA treatment may be protecting dopaminergic neurons from α-syn-related toxicity and degeneration.

### Molecular Modelling Helps to Understand How OA Interacts with α-syn

**Fig. 6:**
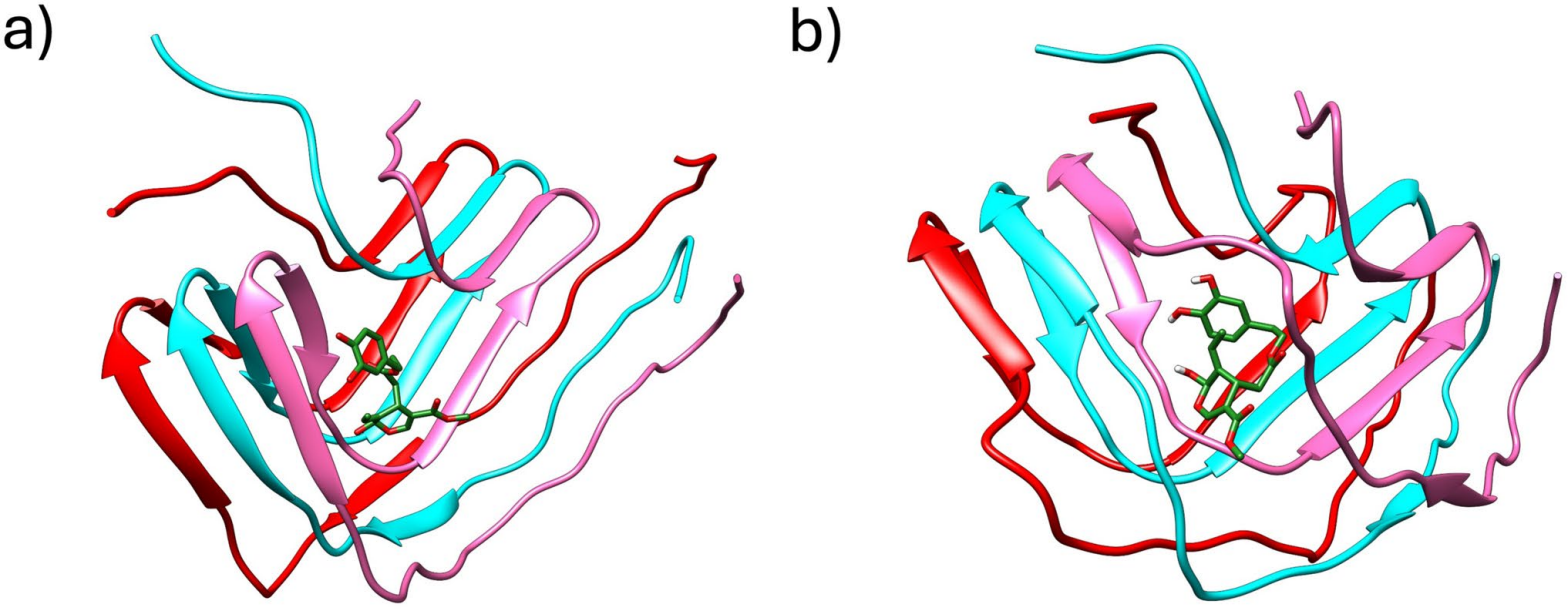
Representatives of T3C1 (**a**) and T4C1 (**b**). Chains are colored red (chain A), cyan (chain B), and pink (chain C). Hydrogens are omitted for clarity

**Fig. 7:**
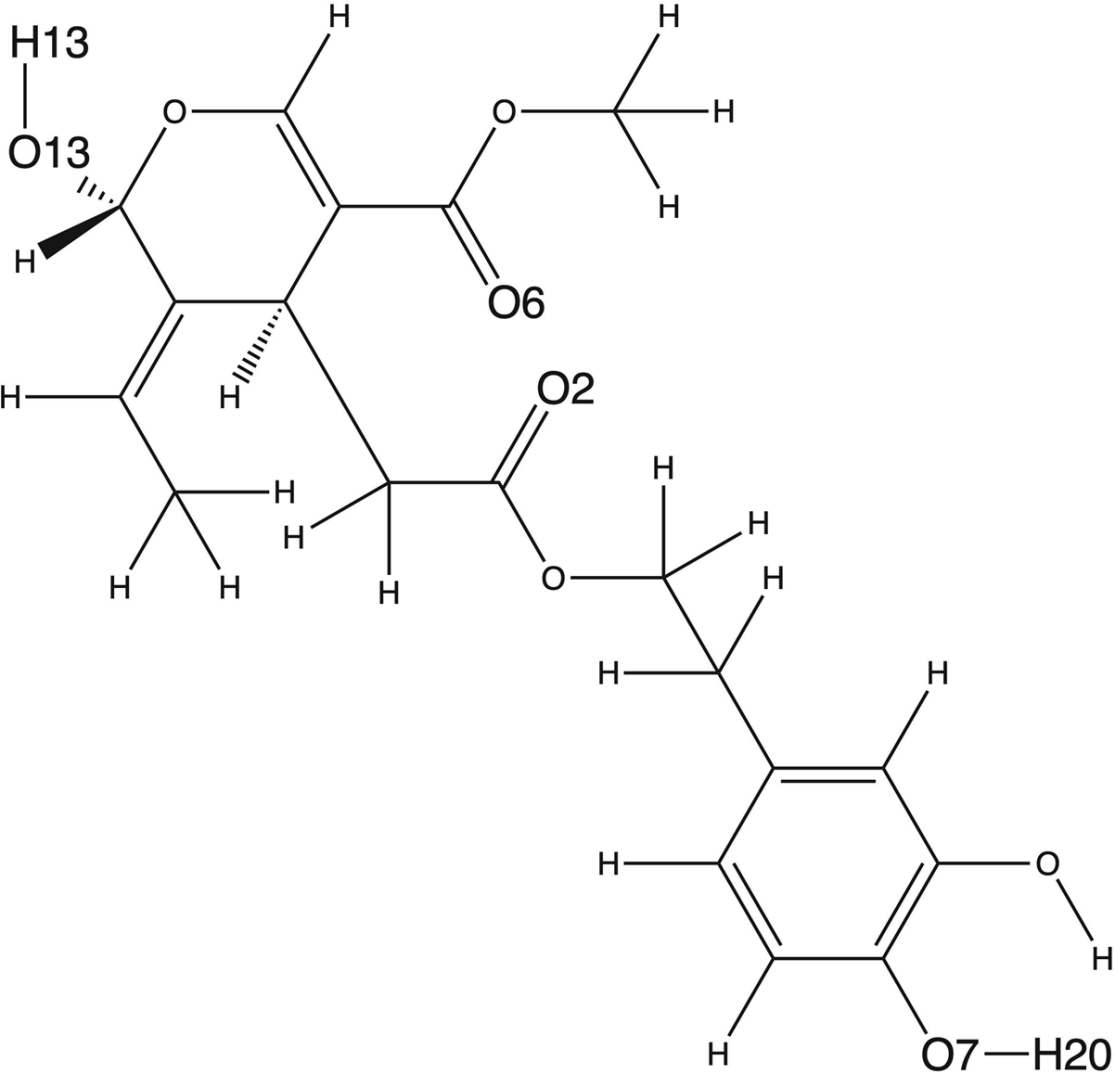
The structure of OA, indicating those atoms involved in h-bonds with the α-syn trimer

A MMGBSA calculation on each of the clusters obtained after the MD run allows to obtain ${\Delta \text{G}}_{\text{binding}}$ for each cluster. These binding free energies, along with the population of each of the clusters, are presented in Table 2. The most favorable binding energies are observed in trimer 3 cluster 1 (T3C1) and trimer 4 cluster 1 (T4C1), with the latter having a predominant population of 95.3% of frames from its MD trajectory, indicating a highly stable binding configuration. Considering both the binding energy and cluster population, we opted to concentrate our analysis on T3C1 and T4C1. Figure 6 depicts representatives of these clusters, while Figure 7S (Supplementary Material S7) shows representatives of the remaining clusters. These figures aid in proposing three distinct interaction modes between OA and the α-syn trimer. Trimer 1 cluster 1 (T1C1) and trimer 2 cluster 1 (T2C1) depict OA interacting with the β-sheet region comprised of residues GLN62-VAL66, without apparent inhibition of aggregation or disruption of the α-syn trimer. Trimer 1 cluster 2 (T1C2), trimer 3 cluster 2 (T3C2), and T3C1 primarily interact with chain C, hindering further growth of the α-syn trimer by recruiting new α-syn chains adjacent to chain C. Finally, T4C1, characterized by the most favorable binding energy and highest population, integrates into the α-syn trimer structure, engaging with both chains B and C, and is anticipated to dismantle the formed fibril.

**Table 2:** Average binding free energy of interaction (${\Delta \text{G}}_{\text{binding}})$ and its corresponding standard deviation ($\sigma$) in kcal mol^−1^, between OA and the different clusters obtained, along with the % of frames composing each cluster

System		${\Delta \mathbf{G}}_{\mathbf{b}\mathbf{i}\mathbf{n}\mathbf{d}\mathbf{i}\mathbf{n}\mathbf{g}}$	${\varvec{\sigma}}$	%Frames
Trimer 1	Cluster 1	−18.07	5.48	37.5
	Cluster 2	−19.64	7.37	21.8
Trimer 2	Cluster 1	−20.84	7.65	39.1
Trimer 3	Cluster 1	−32.86	6.41	49.4
	Cluster 2	−28.91	8.65	25.2
Trimer 4	Cluster 1	−39.15	4.87	95.3

To gain a deeper understanding of the interactions between OA and the α-syn trimer, Table 3 provides insight into the hydrogen bonds formed, along with the percentage of frames in which each hydrogen bond occurs, for the systems analyzed. The atoms of OA shown in Table 3 to establish hydrogen bonds with α-syn can be identified in Figure 7. Notably, OA establishes hydrogen bonds only in T3C1, T3C2, and T4C1. Among these, the hydrogen bonds predicted to be most stable, occurring in 58% of the analyzed snapshots, are formed with GLU61 in T3C1 and T4C1. Furthermore, Table 4 and Table 2S (Supplementary material S8) present the outcomes of a free energy decomposition analysis, revealing that the interactions are primarily of an electrostatic nature, with GLU61 exhibiting the most favorable interaction energy among the amino acids involved.

**Table 3:** Hydrogen bonds established between OA and each α-syn trimer. Only h-bonds established during more than 20% of frames analyzed are shown. Each α-syn trimer chain is labelled in parenthesis as A, B, or C. See Fig. 2 for the atom numbering of OA

**System**	**Acceptor**	**DonorH**	**Donor**	** % Frames**
T3C1	GLU61(B)@OE1,OE2	OLE@H13	OLE@O3	58
	OLE@O2	VAL71(C)@H	VAL71(C)@N	26
T3C2	VAL154@O	OLE@H20	OLE@O7	35
T4C1	VAL71(C)@O	OLE@H20	OLE@O7	57
	OLE@O6	ALA56(C)@H	ALA_139@N	36
	GLU61(B)@OE1,OE2	OLE@H13	OLE@O3	58

**Table 4:** Results of the energy decomposition analysis for the systems T1C1 and T4C1, in kcal mol^−1^. Values for the van der Waals (VDW) electrostatic (ELEC.) and total interaction energy are only shown for residues of the α-syn trimer with a total interaction energy better than kcal mol^−1^. Each α-syn trimer chain is labelled in parentheses as A, B, or C

**System**	**Residue**	**VDW**	**ELEC**	**Total**
T3C1	GLU61(B)	−0.60	−5.96	−5.26
	VAL71(C)	−1.01	−1.83	−3.20
	GLU61(C)	−0.38	−3.77	−3.17
	VAL70(C)	−1.40	−0.79	−3.13
	VAL63(C)	−1.48	−0.27	−2.99
	VAL74(C)	−0.98	−0.34	−2.05
T4C1	GLU61(B)	−1.24	−5.84	−5.90
	VAL74(C)	−2.03	−1.70	−4.49
	VAL71(C)	−0.71	−3.15	−4.16
	VAL115(B)	−1.52	−1.03	−3.32
	ALA56(C)	−1.15	−1.26	−3.00
	THR75(C)	−1.45	−0.05	−2.45
	THR54(C)	−1.27	−0.61	−2.34
	ALA89(B)	−1.08	0.16	−2.01

## Discussion

α-syn aggregation is known to play a pivotal role in the pathogenesis and development of PD and other related synucleinopathies [61]. In this sense, there is a growing interest to search for molecules that prove effective at inhibiting misfolding of this amyloidogenic protein and, subsequently, at preventing the appearance of toxic species (oligomers and protofibrils). Small molecules such as phenolic compounds are among the most studied because of their low toxicity and ability to cross the BBB [62, 63] [64].

Among them, OA has been reported to stabilize the monomeric state of α-syn *in vitro* [23] and to protect against neurodegeneration in transgenic models of Aβ deposition [19]. However, few studies have evaluated OA effects on α-syn aggregation in either cellular or animal models of PD [20, 23]. Thus, in this study, we decided to assess the protective effect of OA on α-syn aggregation and toxicity using different experimental models of PD: (i) two cellular models, namely α-syn overexpressing SK-N-SH cells and α-syn PFF-exposed naive SK-N-SH cells, and (ii) two well-established *C. elegans* strains overexpressing human α-syn.

To date, the capability of OA to reduce α-syn fibrillation and stabilize its monomeric form has been demonstrated in *in vitro*, cell-free systems [21, 23]. One study employed a cellular model (neuroblastoma-derived SH-SY5Y, similar to the one we used) focusing on the toxicity exerted by the downstream products of the α-syn aggregation protocol preformed *in vitro*, namely fibrils, either in the presence or absence of OA [23]. The species of aggregated α-syn that were previously incubated with OA indeed showed a reduced level of toxicity, expressed both as cell viability and ROS production, suggesting that OA can also disrupt preformed synuclein fibrils *in vitro*. In a very similar study, the potential of OA in reducing the formation of proteinaceous aggregates was demonstrated in the same cellular model against preformed Aβ1-42 aggregates [65]. Likewise, the authors exposed cells to end products of *in vitro* preformed amyloid species, grown either in the presence or absence of OA, showing that the aggregates previously incubated with OA were less abundant and less toxic. In our study, we employed for the first time a cell model that naturally features an increase of early α-syn aggregated species due to the overexpression of α-syn [66], and we investigated the extent of α-syn pathology after *on-cell* OA administration. In addition, for the detection of multimers, we took advantage of the PLA technique, which allows the detection of two α-syn molecules in close proximity, providing greater sensitivity to early aggregates compared to total α-syn staining [67]. According to our results, OA exhibited potential in reducing the α-syn burden of a neuronal cell model in a dose-dependent manner. Since these cells already display the presence of multimers before treatment, we can speculate that OA can disrupt at least small aggregates, even after their formation. The slight but significant reduction in total α-syn levels upon OA administration could suggest that the excess of monomeric α-syn deriving from multimers disruption may be cleared by cells with either protein degradation or secretion phenomena, or even by a reduced transcription, possibilities that were not investigated in this study and warrant further investigation. Additionally, we found no positivity for either 5G4 (directed towards β-sheet enriched aggregates [68]) or pS129 antibody (Supplementary figure S4). As these modifications appear to feature late α-syn aggregates [69, 70], we could speculate that, in this model, the overexpression of synuclein only results in the formation of a very early type of aggregates that, without any further insult, stress, or multicellular/systemic contribution, do not evolve into bigger, more toxic, β-sheet enriched aggregates.

After that, we wanted to verify whether OA was able to also clear α-syn greater aggregates. We employed naive SK-N-SH, and we exposed them to *in vitro* preformed α-syn fibrils. Cells were incubated together with either OA or DMSO, showing that OA significantly reduced both size and intensity of α-syn aggregates. In addition, the 3D reconstructions allowed us to reveal the volumetric relationships between PFFs and cell bodies. We observed that smaller α-syn assembly may be internalized within cells, while greater aggregates appeared only partially hooked to the membrane. Interestingly, rather than clearing extracellular aggregates, the administration of OA resulted in a notable decrease of the portion of PFFs that are internalized within cells, either completely or partially. These results could indicate that OA is able, not only to prevent α-syn fibrillation, as suggested by previous *in vitro* studies, but also to clear pre-existent α-syn multimers and bigger aggregates in a neuronal cellular model. In addition, the increased cell viability upon OA administration may indicate a neuroprotective effect against PFF-induced toxicity. Nevertheless, we cannot entirely rule out the possibility that some interaction between OA and PFFs might be occurring in a “cell-free” environment—i.e., the medium—and before PFFs internalization—thus without involving the cells directly or on their surface—especially considering that larger PFFs are not internalized but rather remain attached to cells.

In line with the results obtained in cells, our experiments using *C. elegans* models of PD show a significant inhibition of α-syn accumulation and toxicity *in vivo.* We used the *C. elegans* strain NL5901, a well-established model of PD in *C. elegans*, which has been widely used in many studies to evaluate the effects of different plant extracts and individual compounds on α-syn aggregation [71–73]. This transgenic strain overexpresses human α-syn fused to YFP in the body wall muscle of *C. elegans*, where it, age-dependently, accumulates into inclusions containing aggregated material similar to human pathological inclusions [25]. As a result, these nematodes also exhibit age-dependent mobility defects associated with α-syn aggregation in muscle cells. In a recent study, this PD model was used as proof of concept for the analysis of phase transitions and amyloid conversion of α-syn, supporting the consistency of this specific model to screen for anti-aggregating candidates [74]. In a previous study, we had shown that tyrosol at 1-mM dose was effective in reducing the amount of α-syn inclusions in this strain also increasing longevity [18]. Although a recent work has also shown an inhibitory effect of OA at a similar dose, the *C. elegans* strain used was different and there was no quantification of individual α-syn inclusions [20]. The use of z-stack confocal images allowed us to quantify aggregates in a more accurate way than by total body fluorescence determination, revealing a significant and consistent reduction of α-syn aggregates by 34% compared to non-treated controls. Since OA treatment did not affect overall α-syn expression levels in these nematodes, we can assume that the observed decrease in the amount of α-syn intracellular inclusions may be a consequence of the OA effect on α-syn aggregation in this model. Our results in this strain also showed a slight increase in the thrashing rate of OA-treated nematodes, which may be concomitant with the lower α-syn accumulation in muscular cells observed in response to this treatment.

It has been reported that modulation of protein homeostasis and aggregation pathways has beneficial effects for both health span and lifespan [75]. In this sense, we also observed a positive effect of OA treatment on the *C. elegans* NL5901 strain lifespan. Although we had obtained a similar result with tyrosol in this strain, to our knowledge, OA effect on longevity had not been tested before in any *C. elegans* model of PD. Our results showed that, indeed, OA treatment was able to induce a significant lifespan extension in this strain, which was accompanied by an increased resistance to heat stress. Brunetti et al. evaluated the effect of OA at similar doses on lifespan and stress resistance in *wild-type* nematodes, reporting an improved heat stress survival but no effects on longevity, although these parameters were not evaluated in the *C. elegans* PD strain used in their study [20].

To assess the protective effects of OA on dopaminergic neurons, we used the strain UA44, wherein α-syn expression induces neurodegeneration of anterior and posterior dopaminergic neurons. Our results show that OA treatment was able to significantly delay α-syn-dependent dendrite degeneration as these nematodes age. Other authors have reported a slight effect in the same *C. elegans* strain in response to OA treatment at a similar dose, although the differences were not significant [20].

Although there are very few studies analyzing the role of OA in α-syn aggregation, it has been suggested that OA may interact at different levels with α-syn, promoting the formation of non-toxic off-pathway oligomers that differ from aggregation-prone monomeric forms while hampering the growth of toxic on-pathway oligomers [21, 23]. Although in our study we cannot structurally discriminate between on- and off-pathway aggregates, we could speculate that the improvement of the animal’s lifespan, dopaminergic and motor features, as well as the reduction in the extent of PFFs and the increased viability in cells may indicate a positive effect at least on the toxic, on-pathway aggregation process.

Moreover, at a molecular level, OA has been shown to stabilize the NAC and C-terminal regions of α-syn, preventing the long-range and hydrophobic interactions that favor amyloid aggregation [23]. In this context, we used molecular dynamics simulations to gain insight into the interaction of OA with α-syn and its potential to prevent the formation of amyloid aggregates and inhibit fibrillogenesis.

The molecular modelling results predict three different modes of interaction between OA and the α-syn trimer, although our results point to the most feasible option being OA getting into the trimer structure and interacting with both chains B and C. The proposed mode of interaction would reasonably lead to trimer breakdown, as the main interactions are established with the pre-NAC (residues 47–56) and NAC (residues 61–95) regions, which are essential for thermodynamic stability [76, 77] and for aggregation and cytotoxicity [78, 79]. Thus, our theoretical results support the experimental results in the sense that OA can disrupt small aggregates after their formation. Moreover, MD allows us to know the OA mechanism of action against aggregation at the molecular level, which allows us to propose different pharmacophores for the interaction between the α-syn trimer and OA. These pharmacophores are currently being used to search for molecules with increased affinity for α-syn.

Taken together, our results show that OA treatment can effectively reduce the extent of different types of α-syn aggregates, both in cellular and *C. elegans* models of PD. In addition, both the dopaminergic degeneration upon α-syn overexpression and the cellular toxicity by Syn-PFF administration are recovered after OA administration, thus suggesting that it could exert a neuroprotective effect against pathological α-syn species, as sought in any therapeutic strategy aimed to prevent or treat PD. Moreover, although we cannot rule out other mechanisms, we propose that OA could interact with early α-synuclein oligomers inducing their breakdown, leading to lower cytosolic aggregation of this protein.

## Conclusions

OA treatment reduced early α-syn pathology in SK-N-SH α-syn overexpressing cells in a dose-dependent manner as demonstrated by proximity ligation assay. Moreover, in naive SK-N-SH cells incubated with preformed α-syn fibrils, OA treatment was able to reduce the size and intensity of α-syn aggregates in this neuronal line, also rescuing cell viability upon/against PFF-induced toxicity. By using two different *C. elegans* models of PD, OA treatment at a specific dose resulted in a reduction of α-syn aggregates and toxicity and promoted lifespan and heat stress survival while delaying dopaminergic neurodegeneration. Finally, molecular modelling dynamics propose that OA can interact with specific chains of α-syn trimers, suggesting that primary oligomer breakdown may be one of the mechanisms responsible for the observed reduction of α-syn aggregates in response to OA treatment in both experimental models. Overall, our results support a protective role of OA against α-syn toxic aggregation and shed light on the molecular mechanisms involved, although further studies should be performed to gain insight into the neuroprotective actions of this polyphenol in humans.

## Supplementary Information

Below is the link to the electronic supplementary material.

Supplementary file1 (PDF 100 KB)

Supplementary file2 (DOCX 5604 KB)

## Data Availability

No datasets were generated or analysed during the current study.

## Abbreviations

α-syn: α-synuclein
DA: Dopamine
DMSO: Dimethyl sulfoxide
EVOO: Extra virgin olive oil
FUDR: Fluorodeoxyuridine
LCPO: Linear combinations of pairwise overlaps
MD: Molecular dynamics
MMGBSA: Molecular mechanics general born surface area
OA: Oleuropein aglycone
PD: Parkinson’s disease
PFF: Preformed fibrils
PLA: Proximity ligation assay
