# Supplementary figures and images for: Oleuropein Aglycone, an Olive Polyphenol, Influences Alpha-Synuclein Aggregation and Exerts Neuroprotective Effects in Different Parkinson’s Disease Models
Source: Mol Neurobiol. 2025 Jul 24;62(12):15741–58. doi: 10.1007/s12035-025-05208-6 (PMC12559123; doi:10.1007/s12035-025-05208-6)

Exposure time = 0.5 s

Actin

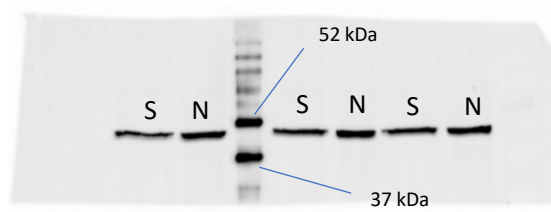

Synuclein

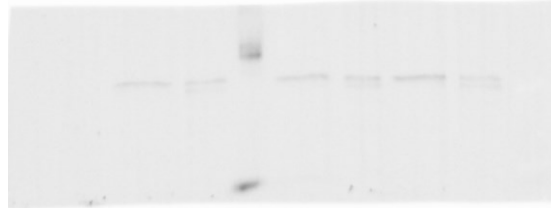

S = Syn+  
N = Naive  
red signal = saturated pixels

Exposure time = 8.5 s

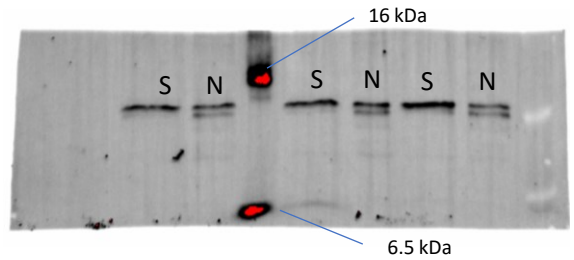

Supplement: Supplementary file 1 — Supplementary file1 (PDF 100 KB) [file 12035_2025_5208_MOESM1_ESM.pdf]
