# Supplementary material for: Oleuropein Aglycone, an Olive Polyphenol, Influences Alpha-Synuclein Aggregation and Exerts Neuroprotective Effects in Different Parkinson’s Disease Models
Source: Mol Neurobiol. 2025 Jul 24;62(12):15741–58. doi: 10.1007/s12035-025-05208-6 (PMC12559123; doi:10.1007/s12035-025-05208-6)
Supplement: Supplementary file 2 — Supplementary file2 (DOCX 5604 KB) [file 12035_2025_5208_MOESM2_ESM.docx]

**Supplementary Materials**

**S1. Western blot**

**
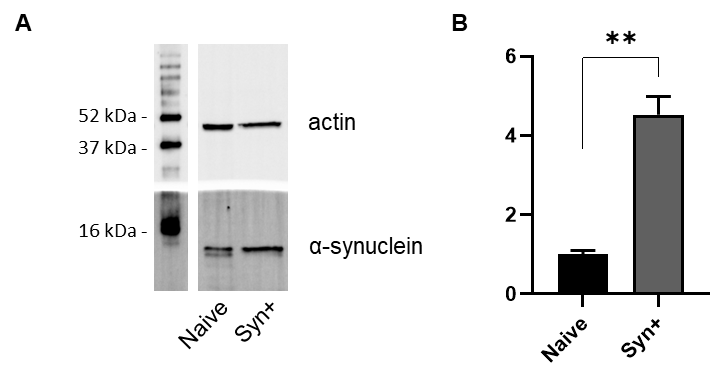
**Total cellular extracts were obtained by scraping using Laemmli buffer (2% SDS, 10% glycerol, 5% β- mercaptoethanol, 0.001% bromophenol blue, and 62.5 mmol/L Tris, pH 6.8) plus protease inhibitors 1:1000 (M250, AMRESCO). Protein concentration was measured with Micro BCA TM Protein Assay Kit (Thermo Scientific). Samples were separated by electrophoresis on bis-tris polyacrylamide gels, then proteins were transferred onto methanol-preactivated PVDF membrane (Immobilon-F transfer membrane, Millipore) by applying constant voltage (60 V) for 90 minutes at 4°C. For α-syn detection, membranes were fixed with paraformaldehyde 0.4% in PBS for 30 minutes. Saturation of non-specific sites was carried out by incubating membranes with a solution of 5% BSA in Tris-buffered saline (TBS) + 0.1% Tween for 1 hour at RT. Then, membranes were incubated ON at 4°C with the following primary antibodies: anti α-syn rabbit IgG antibody (1:1000; S3062, Sigma); anti actin rabbit IgG antibody (1:2000; A2066, Sigma). The incubation with secondary antibodies was performed for 1 hour at RT, in the dark, using the Alexa Fluor 488 donkey anti rabbit antibody (1:4000; Invitrogen, A21206), diluted in in BSA 1% in TBS + Tween 0.1%. Images were acquired at Chemidoc Touch (Biorad) and relative band intensity levels were quantified with ImageLab software (Biorad). The notable increase of α-syn expression in stable transfected SK-N-SH cells is shown in Supplementary figure S1.

**Supplementary Figure S1. α-Synuclein expression levels in stable transfected SK-N-SH. A)** Western blotting for the expression of α-syn and actin. B) Quantification of synuclein levels (relative abundance) in α-syn overexpressing SK-N-SH cells (Syn+) expressed as fold change relative to naive SK-N-SH cells. α-Syn levels in Syn+ cells are approximately 4.5-fold more abundant, compared to naive cells (n=3 biological replicates, p=0.0017 according to Student’s t-test).

**S2. Cytotoxicity assays.**

Oleuropein deglycosylation was successfully carried out, as indicated by the 10 mM glucose concentration measured in the supernatant of the reaction mix. Hoechst test was performed to assess the compound's cytotoxicity, according to manufacturer instructions. SK-N-SH cells were incubated for 48 hours with increasing concentrations of OA, 0.4% DMSO was used as control. The assay indicated that OA does not exert a notable toxic effect, being the percentage of alive cells after 48 hours of treatment close to 90% even at 250 μM, the greatest concentration tested (Supplementary figure S2).


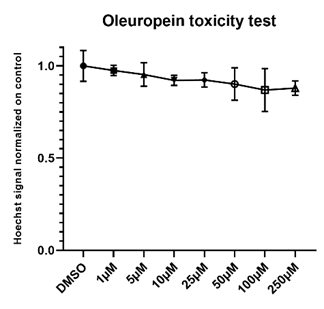


**Supplementary Fig.S2. Hoechst test to assess OA toxicity.** No significant effect on cell viability was observed at the greatest concentration tested. DMSO vs OA 250 μM: p > 0.05 according to Student’s t-test.

**
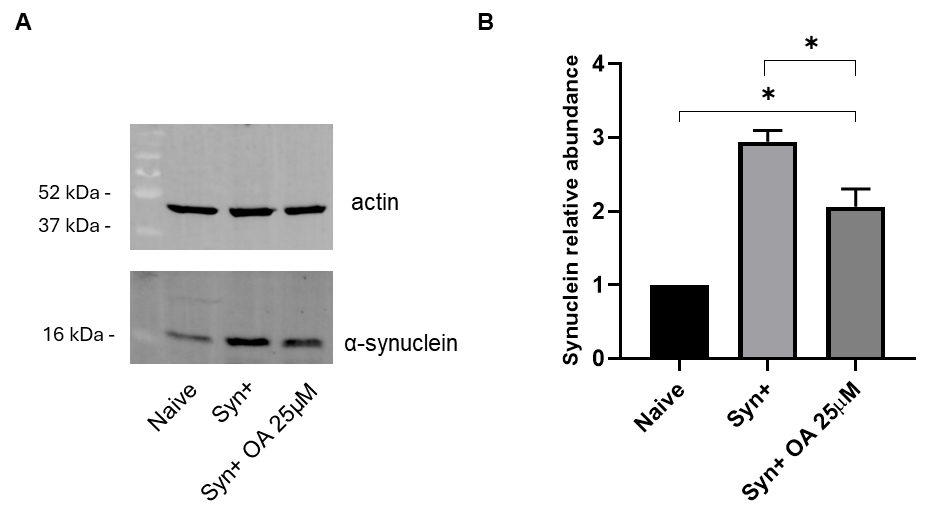
S3. Total α-synuclein expression upon OA administration**

**Supplementary Fig.S3. Total α-synuclein expression upon OA administration**. Western blotting for the expression of α-syn and actin. B) Quantification of synuclein levels (relative abundance) in α-syn overexpressing SK-N-SH cells (Syn+) treated with either OA 25μM or vector only (DMSO 0.1%) for 24 hours, expressed as fold change relative to naive SK-N-SH cells. α-Syn levels in treated cells were reduced by 30%, compared to untreated Syn+ cells, still remaining significantly different (2.06-fold greater) compared to the Naive population (n=3 biological replicates, * p < 0.05 according to One sample t-test).

**S4. Expression of different α-synuclein species**

Immunofluorescence experiments were performed for detecting aggregated (5G4) and phosphorylated synuclein (pS129). The experimental procedure follows the same steps described in section 2.4 of the Methods chapter. Fixed cells were incubated with mixtures of primary antibodies containing the anti total alpha tubulin (302 206, Synaptic Systems, chicken, 1:2000) together with either the mouse anti aggregated α-synuclein 5G4 (clone 5G4, code no. MABN389, 1:250; Merck Millipore), or the rabbit anti Ser129 phosphorylated α-synuclein (clone EP1536Y, code no. ab51253, 1:500, Abcam). The secondary antibodies used were the following: donkey anti-chicken AffiniPure Cy3 (1:300, code 703-165-155, Jackson ImmunoResearch Europe LTD), donkey anti-mouse Alexa Fluor 488 (1:300, code 715-545-151, Jackson ImmunoResearch Europe LTD), donkey anti-rabbit Alexa Fluor 488 (1:300, code A21206, Invitrogen). Nuclei were counterstained with Hoechst 33342. As shown in Supplementary figure S4, , 5G4 antibody provided no positive signal in either Naïve or Syn+ cells. Staining for pS129-synuclein revealed a diffuse pattern in both naïve and Syn+ cells, thus likely to identify the presence of physiological, non-aggregated phospho-synuclein.

**
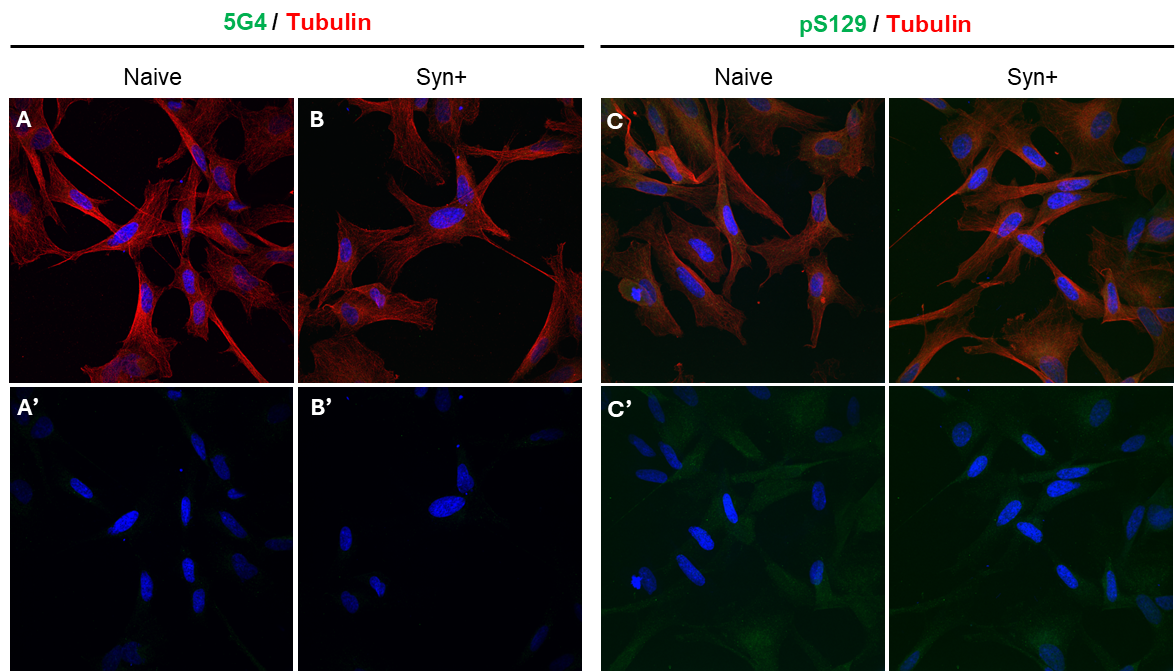
**

**Supplementary Fig.S4. Expression of different α-synuclein species**. Immunostainings for aggregated α-syn (5G4, left panel) and phospho-syn (pS129, right panel) are shown. Total α tubulin is stained in red, nuclei in blue. As seen in A-A’ and B-B’ no positive signal (green) is reported for 5G4 antibody in either Naïve or Syn+ cells. Similarly, no positive signal (green) is reported for pS129 antibody in either Naïve or Syn+ cells (C-C’ and D-D’, respectively). Scale bar, 10 μm.

To evaluate the presence of Thioflavin S positive fibrils, SK-N-SH Syn+ cells were incubated in the presence (as positive control) or absence of α-syn PFFs, and stained according to (*Défossez A, Delacourte A. Transformation of degenerating neurofibrils into amyloid substance in Alzheimer's disease: histochemical and immunohistochemical studies. J Neurol Sci. 1987 Oct;81(1):1-10*). Briefly, samples were incubated with filtered 1% Thioflavin S (Sigma-Aldrich) for 10 min at RT in the dark. Subsequently, sections were washed twice with 80% ethanol for 3 min and with water for 10 min, then mounted with glycerol. The specific staining for Thioflavin S positive fibrils (**Supplementary Fig.S4.1**) is detectable in the presence (A), but not in the absence (B) of α-syn PFFs, indicating that untreated cells do not present fibrils-like inclusions. Contrast phase image was superimposed in A’’- B’’ to highlight cell bodies.

**
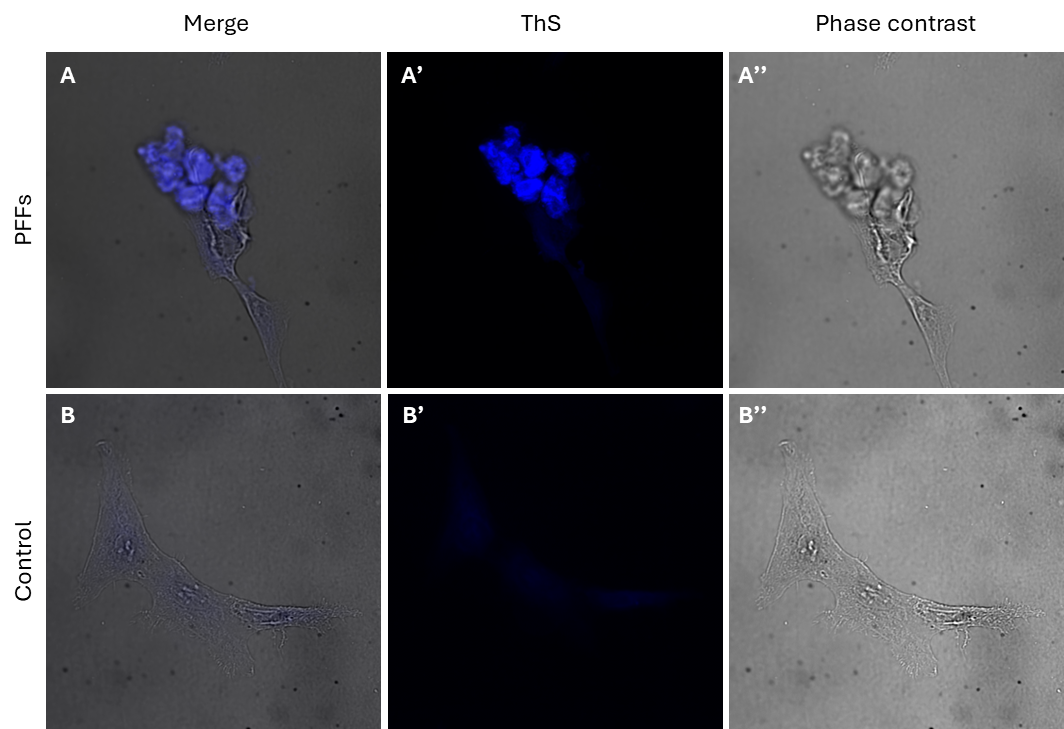

Supplementary Fig.S4.1. Thioflavin S assay for the detection of α-syn fibrillar assemblies.** SK-N-SH Syn+ cells, incubated in the presence (A-A’-A’’) or absence (B-B’-B’’) of α-syn PFFs. Blue signal specifically stains PFFs, and is absent in cell unexposed to aggregates.

**S5. Thioflavin T assay**

PBS, the supernatant containing monomeric α-syn and the PFFs solution underwent ThT assay to evaluate the content of β-sheet structures. Briefly, ThT 10 μM was incubated with either PBS, monomeric α-syn or PFFs in a molarity ratio 1:1, for 10 minutes at RT. Then, fluorescence at 482 nm was read at a multiplate reader. PFFs solution displayed a 12-fold greater beta-sheets concentration compared to PBS alone, and 7.2-fold greater compared to monomeric α-syn as shown in Supplementary Fig.S5.


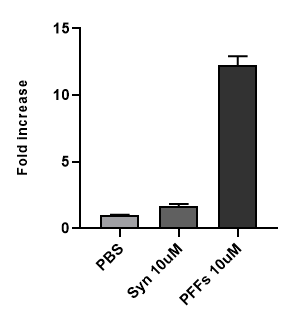

**Supplementary Fig.S5.** β**-Sheets content after 14 days**. Values are expressed as fold increase of the control (PBS).

**S6. Transmission electron microscopy (TEM)**

For electron microscopy, 7 µl of 25 µM protein sample containing α-synuclein PFF was applied to glow discharged carbon formvar copper grids and let dry. Then, negative staining was performed with 1% uranyl acetate, applied twice on the grids for 15 s. Images were acquired with Talos L120C electron microscopy.


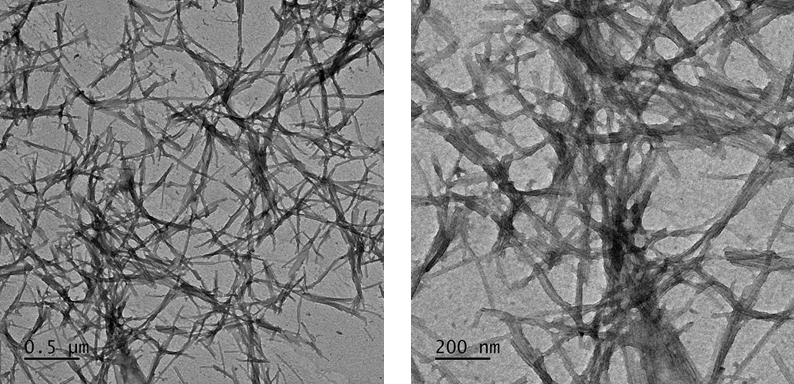

**Supplementary Fig.S6. TEM images of α-synuclein fibrils.** Images obtained after 14 days of incubation. Both fibrils structure and the absence of contamination could be appreciated. Two different magnifications are shown.

**S7. Figure S7.** Representatives of T1C1 (a) and T1C2 (b), T2C1 (c) and T3C2 (d). Chains are colored red (chain A), cyan (chain B) and pink (chain C). Hydrogens are omitted for clarity.


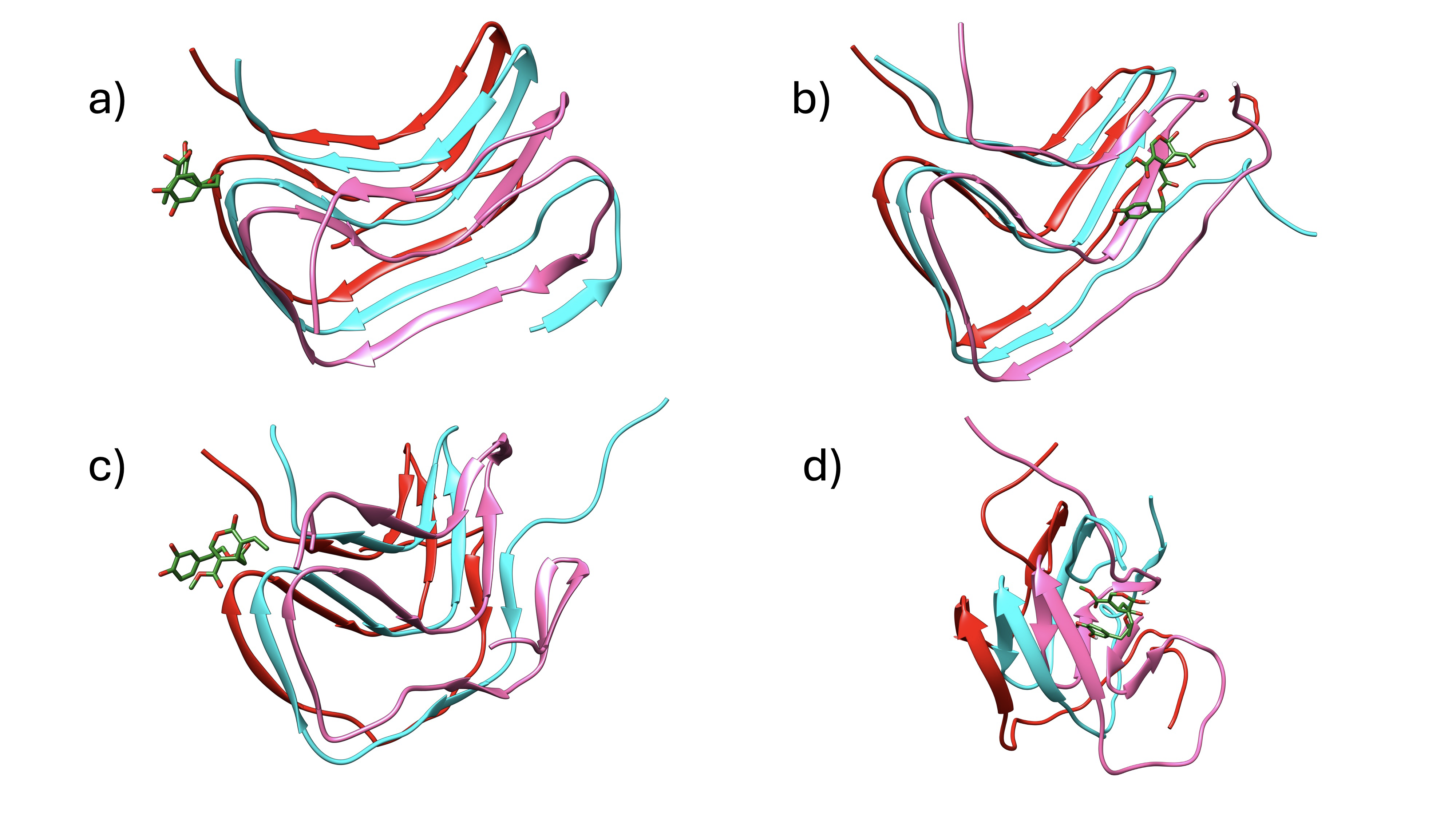


**S8. Table 2S.**

| **System** | **Residue** | **VDW** | **ELEC.** | **TOTAL** |
| --- | --- | --- | --- | --- |
| T1C1 | PHE94(B) | -2.03 | -0.23 | -3.57 |
|  | PHE94(A) | -1.79 | -0.17 | -3.19 |
|  | VAL66(B) | -1.24 | -0.19 | -2.33 |
| T1C2 | TYR39(C) | -1.17 | -0.11 | -2.10 |
| T2C1 | PHE94(B) | -1.83 | -0.81 | -3.79 |
|  | PHE94(A) | -1.72 | -0.17 | -3.03 |
|  | LYS97(B) | 0.11 | -3.03 | -2.71 |
|  | VAL66(B) | -1.16 | -0.18 | -2.18 |
|  | PHE94(C) | -0.96 | -0.41 | -2.00 |
| T3C2 | VAL63(C) | -1.71 | -0.60 | -3.54 |
|  | ILE88(C) | -1.44 | -0.35 | -2.88 |
|  | VAL71(C) | -0.43 | -1.95 | -2.83 |
|  | VAL40(C) | -0.78 | -1.02 | -2.29 |
|  | VAL70(C) | -1.05 | -0.58 | -2.26 |
|  | ALA89(C) | -1.14 | -0.35 | -2.19 |
|  | GLU61(C) | -0.71 | -1.32 | -2.18 |
|  | GLU61(B) | -0.36 | -1.48 | -2.18 |
|  | LYS97(B) | -0.34 | -2.37 | -2.15 |

Table 2S. Results of the energy decomposition analysis for the systems T1C1, T1C2, T2C1 and T3C2, in kcal mol^-1^. Values for the van der Waals (VDW) electrostatic (ELEC.) and total interaction energy are only shown for residues of the α-syn trimer with interaction a total interaction energy better than -2 kcal mol^-1^. Each α-syn trimer chains are labelled in parenthesis as A, B, or C.

**S 9. Preparation of worm protein extracts and western blotting**

For protein extraction, 20 worms per sample were manually collected at the 5^th^ day of adulthood and pooled in 15 μL of lysis buffer (Urea 7M, Thiourea 2M, CHAPS 4%, PMSF 1 mM, DTT 20 mM, pH 8.5). After freezing in liquid nitrogen, 5 μL of Laemli Buffer 4X (BioRad) was added and the mix was heated at 95 °C for 10 minutes. Three biological replicates were prepared for each experimental condition. Proteins were separated by SDS-PAGE in a 12% polyacrilamide gel (BioRad) and transferred to Immobilon P PVDF membranes (Millipore). For α-syn:YFP detection, blots were probed with anti α-syn monoclonal (Invitrogen; 1:2000 dilution) followed by anti-mouse secondary antibody (Sigma-Aldrich) at a 1:5000 dilution. Actin was used as loading control. ECL Prime kit (GE Healthcare Life Sciences) was used for signal detection, following manufacturer's instructions. Images were acquired at Odyssey XF Imager (LICORbio) and relative band intensity levels were quantified with ImageJ software (National Institutes of Health, Bethesda, MD, USA). The results of α-syn expression are shown in Supplementary Figure S9.

**Supplementary Figure S9. α-Synuclein expression levels in NL5901 nematodes in response to OA treatment.** Western blotting for the expression of α-syn:YFP and actin in Control and 1 mM OA treated NL5901 nematodes. All lanes were loaded with equal protein extract from synchronized adult NL5901 worms. Actin was used as loading control. No significant differences in the total amount of α-syn:YFP were found. A) α-syn:YFP levels detected with anti α-syn antibody in *C. elegans* extracts after 5 days of OA treatment. B) Quantification of α-syn levels (relative abundance) in OA treated nematodes relative to vehicle treated controls (n=3 biological replicates, p>0.05 according to Student’s *t*-test).
